# Prevalence, Predictors, and Clinical Outcomes of Cervical Arterial Dissection in Patients with Spontaneous Coronary Artery Dissection: A Multicenter Retrospective Cohort Study

**DOI:** 10.3390/jcm15135302

**Published:** 2026-07-07

**Authors:** Hend Bcharah, Hussein Abdul Nabi, Luke Dreher, George Bcharah, Katie Mand, Vinicius De Sousa Barzon Serra, Linnea M. Baudhuin, Yuxiang Wang, Mayowa A. Osundiji, Fadi E. Shamoun

**Affiliations:** 1 Department of Cardiovascular Disease, Mayo Clinic Hospital, Phoenix, AZ 85054, USA; bcharah.hend@mayo.edu (H.B.); abedalnabihussein@gmail.com (H.A.N.); dreher.luke@mayo.edu (L.D.); bcharahgeorge@gmail.com (G.B.); mand.katie@mayo.edu (K.M.); desousabarzonserra.vinicius@mayo.edu (V.D.S.B.S.); baudhuin.linnea@mayo.edu (L.M.B.); wang.yuxiang@mayo.edu (Y.W.); osundiji.mayowa@mayo.edu (M.A.O.); 2 Department of Clinical Genomics, Mayo Clinic Hospital, Phoenix, AZ 85054, USA

**Keywords:** spontaneous coronary artery dissection (SCAD), cervical artery dissection (CvAD), stroke, SCAD recurrence, all-cause mortality

## Abstract

**Background**: Patients with spontaneous coronary artery dissection (SCAD) are at increased risk of cardiovascular events, including dissection in other arterial territories such as cervical arteries. Those with combined coronary and cervical arterial dissection (CACAD) may have more widespread arterial abnormalities and worse outcomes, underscoring the need for better risk stratification and personalized care. **Objective**: This study examined the prevalence of cervical artery dissection (CvAD) in patients diagnosed with SCAD, evaluated potential risk factors for developing CvAD, and assessed their impact on clinical outcomes, including all-cause mortality, SCAD recurrence, and stroke. The goal was to enhance risk stratification by identifying SCAD patients at increased risk for CvAD. **Design**: In this retrospective cohort study, patients diagnosed with SCAD between 2018 and 2024 across Mayo Clinic sites were identified using ICD-10 codes. Manual chart review confirmed CvAD diagnoses. Patients were stratified based on the presence or absence of CvAD. Chi-square and independent t-tests were used to compare risk factors and comorbidities. Univariate screening and multivariable logistic regression were used to identify predictors of CvAD. **Results**: Among 1380 patients with SCAD, 131 (9.5%) had CvAD. The median follow-up was 2.02 years (IQR: 1.1–5.2 years). All CvAD diagnoses were identified after the index SCAD event through systematic post-SCAD vascular imaging. The mean age was similar between groups (51.5 ± 11.3 years in CACAD vs. 50.9 ± 10.7 years in SCAD-only, *p* = 0.540), and the majority were female (93.9% vs. 91.9%, *p* = 0.529). Multivariable regression identified extremity arterial dissection (OR: 8.13, 95% CI: 3.41–19.36, *p* < 0.001), fibromuscular dysplasia (FMD) (OR: 7.52, 95% CI: 4.67–12.10, *p* < 0.001), connective tissue disorders (CTDs) (OR: 6.85, 95% CI: 2.35–19.98, *p* < 0.001), and anxiety (OR: 1.69, 95% CI: 1.01–2.81, *p* = 0.044) as significant predictors of CvAD. CvAD was a strong independent predictor of stroke (OR: 6.37, 95% CI: 2.93–13.87, *p* < 0.001). Mortality and SCAD recurrence did not differ significantly between groups. **Conclusions**: Patients with CACAD demonstrate a systemic vascular phenotype with significantly higher rates of FMD, connective tissue disease, extremity arterial dissection, and stroke. These findings highlight the importance of comprehensive vascular screening and individualized monitoring in at-risk SCAD patients.

## 1. Introduction

Spontaneous coronary artery dissection (SCAD) is an infrequent but an increasingly recognized cause of acute coronary syndrome (ACS), which results from a tear in the coronary artery wall that creates a false lumen and restricts blood flow, potentially leading to myocardial infarction [1,2]. SCAD disproportionally affects younger women, accounting for 1–4% of all ACS cases and up to 35% of myocardial infarctions in women under age 50 [1]. It is often associated with fibromuscular dysplasia (FMD) and connective tissue disorders (CTDs) [1]. Recognition of SCAD has improved due to advances in intracoronary imaging and clinical awareness [3].

A strong association has been documented between SCAD and FMD, a non-atherosclerotic arteriopathy characterized by abnormal fibrous tissue and smooth muscle proliferation [4]. FMD leads to structural arterial fragility and is found in up to 60% in patients with SCAD, often affecting multiple vascular beds such as renal, carotid, and vertebral arteries [5,6,7]. These findings suggest a shared vascular vulnerability that extends beyond coronary circulation. Recent genetic studies further support this hypothesis by identifying potential hereditary patterns among SCAD and FMD patients, emphasizing the relevance of genetic testing in patients with widespread arterial abnormalities [8].

Cervical artery dissection (CvAD), which involves the internal carotid and vertebral arteries, is another form of spontaneous dissection that can cause ischemic events, particularly in younger individuals, with an annual incidence of 2.5–3 per 100,000 [9]. CvAD has also been associated with CTDs and FMD, indicating potential overlap in underlying arteriopathies [9,10]. A subset of patients may experience both SCAD and CvAD (referred to here as CACAD), but risk factors predisposing to this overlap remain poorly defined. Previous research has shown that SCAD patients have higher rates of extracoronary vascular abnormalities, including arterial tortuosity and aneurysms [11,12,13]. These findings are supported by recent large-scale registry studies that highlight the systemic nature of vascular abnormalities in SCAD, including both structural and genetic contributors [14]. However, it is not well understood which SCAD patients are at risk for CvAD, and whether this dual vascular involvement reflects a more diffuse or syndromic arteriopathy. Addressing this gap is critical for tailoring diagnostic workup and long-term monitoring.

## 2. Materials and Methods

### 2.1. Study Population

This retrospective cohort study included all patients diagnosed with spontaneous coronary artery dissection (SCAD) between January 2018 and December 2024 across Mayo Clinic sites in Rochester, Arizona, and Florida. Patients were identified using the International Classification of Diseases, Tenth Revision (ICD-10) code I25.42. A chart review was conducted to confirm the diagnosis of SCAD and to identify and exclude individuals with non-spontaneous coronary artery dissection. Patients were then stratified into two groups: those with concomitant cervical artery dissection (CACAD) and those with SCAD only.

Inclusion criteria were: (1) patients aged ≥18 years with a diagnosis of SCAD confirmed by coronary angiography, intravascular ultrasound, or optical coherence tomography; (2) ICD-10 code I25.42 recorded between January 2018 and December 2024 at Mayo Clinic sites in Rochester (Minnesota), Scottsdale/Phoenix (Arizona), or Jacksonville (Florida); and (3) SCAD diagnosis confirmed by manual chart review. Exclusion criteria were: (1) non-spontaneous coronary artery dissection (iatrogenic or procedure-related dissection identified on chart review); (2) atherosclerotic coronary artery dissection; and (3) traumatic coronary artery dissection.

Extremity arterial dissection was defined as spontaneous dissection of a peripheral limb artery, including the iliac, femoral, popliteal, brachial, radial, or ulnar arteries. This definition explicitly excluded coronary artery dissection (the index event) and cervical artery dissection (the outcome variable) to avoid circular ascertainment.

A centralized database was constructed using the Mayo Clinic Data Explorer Tool, which was used to extract demographic variables, clinical characteristics, and comorbid conditions. Connective tissue disorder (CTD) features were identified using ICD-10 codes and included mitral valve prolapse (MVP), spontaneous pneumothorax, scoliosis or kyphosis, pectus deformities (excavatum or carinatum), joint conditions (pain, ligament sprain, stiffness, recurrent dislocations, tendon rupture), hypermobility, valgus or varum deformities, varicose veins, flat feet (pes planus), hammer toes, spontaneous organ perforation and herniation, dermatologic features (e.g., atrophic striae, hyperelastic or redundant skin), and postural orthostatic tachycardia syndrome (POTS). Additional manual chart review was conducted to confirm cervical artery dissection diagnoses. The presence or absence of aortic dilation was determined from available echocardiographic data using patient-specific reference ranges. Extracoronary vascular abnormalities, including fibromuscular dysplasia by vascular territory, non-coronary dissections, and aneurysms, were systematically assessed from available cross-sectional imaging.

### 2.2. Study Analysis and Outcomes

Baseline demographic and clinical characteristics, including comorbidities, were compared between SCAD patients with and without CvAD. The prevalence of extracoronary vascular abnormalities, including FMD by vascular territory, non-coronary dissections, aneurysms, and aortic dilation, was assessed. Primary clinical outcomes included stroke, all-cause mortality, and SCAD recurrence.

### 2.3. Statistical Analysis

Nominal variables were summarized as counts and percentages, while continuous variables were expressed as mean ± standard deviation (SD). Categorical variables were compared using chi-square tests, and continuous variables were compared using independent t-tests. Univariate logistic regression was used to assess associations between risk factors and outcomes. Variables with *p* < 0.20 or deemed clinically significant were included in multivariable logistic regression models. Separate multivariable models were also constructed for four outcomes: CvAD, stroke, all-cause mortality, and SCAD recurrence. Results are reported as odds ratios (ORs) with 95% confidence intervals (CIs). A *p*-valve less than 0.05 was considered statistically significant.

## 3. Results

### 3.1. Baseline Characteristics and Comorbidities

**Figure 1:**
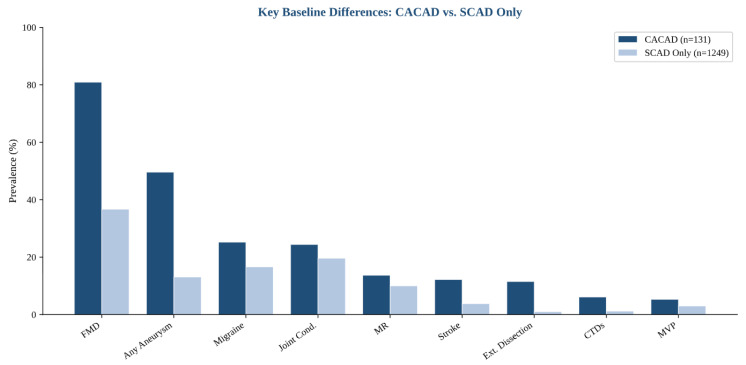
Key Baseline Differences in CACAD vs. SCAD-Only. Bar chart comparing prevalence of key clinical features between CACAD and SCAD-only groups (all differences *p* < 0.05).

A total of 1380 patients were diagnosed with SCAD, of whom 131 (9.5%) had cervical artery dissection (CvAD). Baseline characteristics of the CACAD and SCAD-only groups are summarized in Table 1 (Figure 1). The mean age was 51.5 ± 11.3 years in the CACAD group and 50.9 ± 10.7 years in the SCAD-only group (*p* = 0.540). The proportion of female patients was 93.9% in the CACAD group versus 91.9% in the SCAD-only group (*p* = 0.529). Median follow-up for the cohort was 2.02 years (IQR: 1.1–5.2 years). All 131 cervical arterial dissections in the CACAD group were identified after the index SCAD event through systematic vascular imaging performed as part of the institutional screening protocol. While some dissections may have antedated the SCAD event but remained clinically occult, all CvAD diagnoses were made during the post-SCAD imaging workup.

Patients with CACAD had significantly higher rates of fibromuscular dysplasia (80.9% vs. 36.7%, *p* < 0.001), migraine (25.2% vs. 16.6%, *p* = 0.019), connective tissue disorders (6.1% vs. 1.2%, *p* < 0.001), extremity arterial dissection (11.5% vs. 1.0%, *p* < 0.001) (Table 1). CACAD patients also had significantly higher rates of renal FMD (48.1% vs. 23.2%, *p* < 0.001), iliac/femoral FMD (31.3% vs. 12.7%, *p* < 0.001), and any aneurysm (49.6% vs. 13.1%, *p* < 0.001) (Table 2).

Dilated aorta did not differ significantly between groups (23.4% vs. 20.0%, *p* = 0.520). Stroke was more common in the CACAD group (12.2% vs. 3.8%, *p* < 0.001). Rates of hypertension, hyperlipidemia, diabetes, heart failure and other baseline cardiovascular risk factors were similar between groups (Table 1).

**Figure 2:**
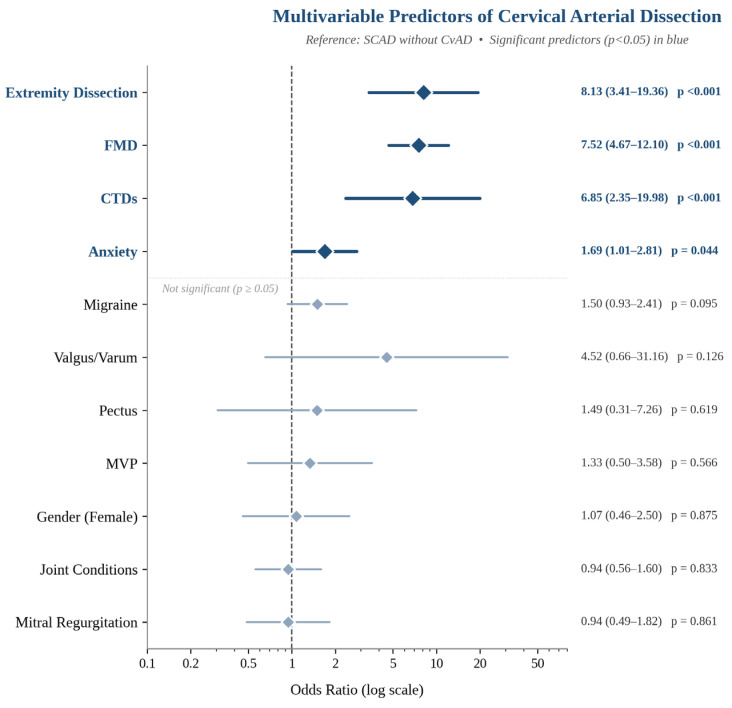
Multivariable Predictors of CvAD. Model: *n* = 1380; 131 events; pseudo-R^2^ = 0.170; model *p* < 0.001.

Univariate logistic regression screening identified 10 variables meeting the *p* < 0.20 threshold (Supplemental Table S1). Multivariable logistic regression identified extremity arterial dissection (OR: 8.13, 95% CI: 3.41–19.36, *p* < 0.001), FMD (OR: 7.52, 95% CI: 4.67–12.10, *p* < 0.001), CTDs (OR: 6.85, 95% CI: 2.35–19.98, *p* < 0.001), and anxiety (OR: 1.69, 95% CI: 1.01–2.81, *p* = 0.044) as significant independent predictors of CACAD. Migraine trended toward significance (OR: 1.50, 95% CI: 0.93–2.41, *p* = 0.095) (Table 3) (Figure 2).

### 3.2. Outcomes

Patients with CACAD had higher rates of stroke (12.2% vs. 3.8%, *p* < 0.001). Multivariable regression analysis identified CvAD as a strong predictor of stroke (OR: 6.37, 95% CI: 2.93–13.87, *p* < 0.001), along with obesity (OR: 2.68, 95% CI: 1.31–5.52, *p* = 0.007) and atrial fibrillation (OR: 2.67, 95% CI: 1.20–5.92, *p* = 0.016). FMD was inversely associated with stroke after adjustment (OR: 0.38, 95% CI: 0.18–0.77, *p* = 0.007) (Table 4).

Mortality was rare overall (1.1%) and did not differ significantly between groups (CACAD 1.5% vs. SCAD-only 1.0%, *p* = 0.646). CvAD was not an independent predictor of death after adjustment (OR: 2.99, 95% CI: 0.47–18.92, *p* = 0.245). SCAD recurrence rates were also similar between groups (46.6% for CACAD vs. 44.4% for SCAD-only, *p* = 0.695), and CvAD was not independently associated with recurrence (OR: 0.87, 95% CI: 0.59–1.29, *p* = 0.483) (Table 5; full models shown in Supplemental Tables S2 and S3).

## 4. Discussion

In this multicenter retrospective study of 1380 patients with SCAD, nearly one in ten had concomitant cervical artery dissection. These patients exhibited a distinct vascular profile characterized by higher rates of FMD, CTD, extremity arterial dissection, and aneurysms that point toward a diffuse, systemic process rather than an isolated coronary event. CvAD was also independently associated with a sixfold increase in stroke risk, while all-cause mortality and SCAD recurrence were similar between groups. These findings raise important considerations regarding vascular screening and long-term surveillance in this patient population.

The prevalence of CvAD in this cohort is overall consistent with prior reports. A study on 214 patients with SCAD identified cervical dissections or pseudoaneurysms in 13.1% of patients. On the other hand, the iSCAD registry reported a lower rate of extracoronary dissections, although cervical arteries were not imaged in all patients in the cohort [14]. These differences in reported prevalence across studies are likely due to variations in cohort size, imaging protocols, and the completeness in cervical vascular assessment. Regardless, these rates signify that CvAD may not be a rare occurrence in a SCAD patient. More importantly, those with CvAD had significantly higher rates of FMD across multiple arterial beds, more frequent aneurysms, and a greater burden of connective tissue features. This pattern suggests that CvAD may serve as a marker of an extensive underlying arterial disease, rather than an isolated finding.

FMD was the strongest independent predictor of CvAD (OR: 7.52, 95% CI: 4.67–12.10) and was present in 80.9% of CACAD patients compared with 36.7% of the SCAD-only group. This finding reinforces the established link between FMD and diffuse arterial disease [6,7]. The CACAD group had significantly higher rates of FMD involvement across multiple arterial beds, including renal and iliac/femoral arteries. Whether the vascular fragility observed in CACAD patients can be attributed solely to FMD or reflects a broader, incompletely characterized arteriopathy warrants consideration. Around 19% of CACAD patients did not carry an FMD diagnosis, yet were found to have multiple dissections, aneurysms, or both. This is also consistent with prior reports of extracoronary vascular abnormalities in the absence of FMD [1,15]. Genome wide association studies have identified genetic loci shared by both SCAD and FMD, including PHACTR1 and LRP1 [16,17,18]. However, other loci appear to be SCAD-specific (ADAMTSL4), which has been associated with SCAD across multiple genome-wide association studies but has not been identified in FMD [16,17,18]. The partial genetic overlap between SCAD and FMD supports the concept that SCAD may belong to a diffuse arteriopathy, with FMD as the most common but not the sole driver of disease. The association of migraine with both SCAD and CvAD may reflect a vascular endothelial dysfunction that is independent from FMD [19,20]. Overall, this data suggests that a subset of SCAD patients may have an underlying systemic arteriopathy that predisposes them to diffuse vascular fragility.

The sex and gender dimension of this cohort merits specific consideration. Over 92% of patients in both the CACAD and SCAD-only groups were female, consistent with the well-established female predominance in SCAD, which accounts for 24–35% of myocardial infarctions in women under 60 years of age [21]. SCAD is increasingly recognized as a leading etiology of Type 2 myocardial infarction in women, with sex-specific hormonal, vascular, and psychosocial contributors driving its pathogenesis [22]. Estrogen fluctuations modulate endothelial function, nitric oxide bioavailability, and matrix metalloproteinase activity, and the postmenopausal decline in estrogen has been linked to coronary vasospasm, microvascular dysfunction, and alterations in extracellular matrix turnover that may predispose to arterial dissection [21]. Notably, pregnancy-associated SCAD, the most common cause of myocardial infarction during pregnancy, occurs predominantly in the postpartum period when estrogen levels decline sharply, further implicating hormonal withdrawal in arterial wall vulnerability [21]. The predominance of women in our CACAD subgroup raises the possibility that the diffuse arteriopathy observed in these patients, including multiterritorial FMD, cervical dissection, and extremity arterial involvement, may reflect a sex-specific vascular fragility mediated in part by hormonal and microvascular mechanisms. Women also demonstrate heightened susceptibility to emotional stress-induced vascular injury, with a two-fold higher risk of mental stress-induced myocardial ischemia and up to ten-fold higher risk of Takotsubo syndrome compared to men [23]. Although the small proportion of male patients in both groups (approximately 6–8%) precluded a formal sex-stratified analysis of predictors or outcomes, future studies with larger male representation are needed to determine whether the CACAD phenotype differs by sex and whether sex-specific hormonal or vascular mechanisms drive the systemic arteriopathy observed in this subgroup.

This analysis also identified two novel independent predictors of CvAD that, to our knowledge, have not been previously reported. Extremity arterial dissection was a strong predictor (OR: 8.13, 95% CI: 3.41–19.36), present in 11.5% of CACAD patients and 1.0% of SCAD-only patients). Anxiety was also an independent predictor of CACAD (OR: 1.69, 95% CI: 1.01–2.81). Anxiety and depression are prevalent among SCAD patients, and emotional stress has been identified as a trigger for SCAD [24]. Arterial wall shear stress and predisposition to dissection in structurally vulnerable vessels may increase as a result of anxiety-related sympathetic activation. The mechanistic basis for this association likely involves the neuroendocrine stress axis. Chronic anxiety activates the hypothalamic–pituitary–adrenal (HPA) axis and the sympathetic nervous system, resulting in sustained elevations of cortisol and catecholamines that promote endothelial dysfunction, arterial wall remodeling, and increased shear stress on structurally vulnerable vessels [23,25]. Notably, these stress responses exhibit important sex-dependent differences: women demonstrate heightened glucocorticoid reactivity, greater locus coeruleus-noradrenaline system sensitivity, and a two-fold higher risk of mental stress-induced myocardial ischemia compared to men [23]. The predominantly female composition of our cohort raises the possibility that anxiety-related sympathetic surges may have a disproportionate vascular impact in women, particularly in those with an underlying arteriopathy [25]. Whether anxiety in our cohort represents a true independent risk factor, a marker of heightened sympathetic tone in susceptible individuals, or a consequence of living with underlying vascular disease warrants further investigation in prospective studies with structured psychological assessment.

The inverse association between FMD and stroke after multivariable adjustment (OR: 0.38, 95% CI: 0.18–0.77, *p* = 0.007) warrants explicit discussion. This counterintuitive finding likely reflects surveillance and treatment bias rather than a true protective biological effect. Patients with a known FMD diagnosis typically undergo more systematic vascular surveillance, including dedicated cervical and intracranial imaging, and are more likely to receive antiplatelet therapy for secondary prevention [5,7]. This heightened clinical vigilance may lead to earlier detection of subclinical vascular abnormalities and prompt initiation of preventive measures that reduce subsequent stroke risk. Additionally, FMD-diagnosed patients may be counseled more aggressively on modifiable risk factors and activity modifications. This ascertainment-treatment bias, in which the diagnosis itself triggers interventions that alter outcomes, is a recognized phenomenon in observational studies of vascular conditions. Alternatively, the FMD coefficient may partially capture confounding from unmeasured variables that correlate with both FMD diagnosis and lower stroke risk. This finding should be interpreted as hypothesis-generating and merits prospective validation.

The findings of this study have several clinical implications. The prevalence of CvAD in our cohort reinforces current recommendations for comprehensive vascular imaging in patients with SCAD [2,26]. Our study suggests that the co-presence of FMD, CTD, extremity arterial dissection, or migraine may help identify high risk patients who may benefit from prioritized or frequent cervical assessment. The association between CvAD and stroke reinforces the importance of early detection and allows for implementation of secondary prevention. Comprehensive vascular assessment, including evaluation for aneurysms, extremity arterial dissections, and connective tissue features may identify patients at risk of diffuse vascular disease, despite the absence of an FMD diagnosis. Several specific research priorities emerge from these findings. First, implementation of standardized cervical vascular imaging protocols in all SCAD patients, regardless of neurological symptoms, would enable more accurate prevalence estimation and earlier detection of CvAD. Second, systematic genetic testing targeting loci implicated in both SCAD and FMD, including PHACTR1, LRP1, and the SCAD-specific ADAMTSL4 locus [16,17,18], may identify patients harboring a genetic predisposition to diffuse arteriopathy. Third, prospective longitudinal registries incorporating structured anxiety and psychological stress assessment, comprehensive vascular imaging, and sex-stratified analyses are needed to clarify the role of emotional stress as a modifiable risk factor and to define the natural history of CACAD over time.

### Limitations

This study has several limitations. As a retrospective analysis limited to three Mayo Clinic sites, selection bias and limited generalizability are possible. Diagnostic coding (ICD-10) was used to identify conditions, which may result in misclassification despite supplemental chart review. Aortic measurements were drawn from the most recent echocardiograms, regardless of timing relative to SCAD or CvAD events, which may affect associations. CTD features were based on diagnostic codes without uniform criteria for formal CTD classification, potentially introducing heterogeneity. Additionally, data on the completeness of head-to-pelvis vascular imaging was inconsistent, which may have led to underestimation of aneurysms or other vascular abnormalities. Future prospective studies with standardized imaging and genetic evaluation are needed to better characterize systemic arteriopathy in patients with SCAD and CvAD. We note that all CTD features were confirmed by manual chart review rather than relying solely on automated ICD-10 code extraction; however, the small number of patients with CTD features (*n* = 23) precluded a sensitivity analysis restricted to formally diagnosed connective tissue disorders (e.g., Ehlers-Danlos syndrome or Marfan syndrome). Additionally, patients with a known diagnosis of fibromuscular dysplasia may have undergone more intensive and comprehensive vascular imaging as part of their clinical care, potentially increasing the likelihood of detecting cervical artery dissection and aneurysms in this subgroup. This surveillance bias may have contributed to the observed association between FMD and CACAD and should be considered when interpreting the strength of this relationship.

## 5. Conclusions

This study suggests that patients with concomitant cervical and coronary artery dissection (CACAD) represent a distinct subgroup of patients with SCAD who exhibit a higher burden of systemic vascular abnormalities. These include significantly greater rates of fibromuscular dysplasia, connective tissue features, extremity arterial dissection, and stroke. While SCAD recurrence and mortality were numerically higher in CACAD, these differences were not statistically significant. Our findings support the hypothesis that a subset of patients with SCAD may have an underlying systemic arteriopathy, possibly syndromic in nature. Future prospective studies incorporating comprehensive imaging and genetic analysis are warranted to improve risk stratification and guide management in this higher-risk population.

## Figures and Tables

**Table 1:** Baseline Characteristics of the Overall Cohort.

Variable	All Patients (*n* = 1380)	CACAD (*n* = 131)	SCAD-Only (*n* = 1249)	*p*-Value
Demographics				
Age, years (mean ± SD)	50.9 ± 10.8	51.5 ± 11.3	50.9 ± 10.7	0.540
Female	1271 (92.1%)	123 (93.9%)	1148 (91.9%)	0.529
White race	1243 (90.1%)	121 (92.4%)	1122 (89.8%)	0.845
Comorbidities				
Hypertension	470 (34.1%)	43 (32.8%)	427 (34.2%)	0.829
Hyperlipidemia	516 (37.4%)	52 (39.7%)	464 (37.1%)	0.633
Diabetes mellitus	79 (5.7%)	5 (3.8%)	74 (5.9%)	0.429
Obesity	151 (10.9%)	11 (8.4%)	140 (11.2%)	0.404
Migraine	240 (17.4%)	33 (25.2%)	207 (16.6%)	0.019
Atrial fibrillation	76 (5.5%)	8 (6.1%)	68 (5.4%)	0.908
Heart failure	223 (16.2%)	20 (15.3%)	203 (16.3%)	0.867
Thyroid disorders	179 (13.0%)	13 (9.9%)	166 (13.3%)	0.340
Depression	198 (14.3%)	22 (16.8%)	176 (14.1%)	0.479
Anxiety	297 (21.5%)	36 (27.5%)	261 (20.9%)	0.103
Vascular				
Fibromuscular dysplasia	564 (40.9%)	106 (80.9%)	458 (36.7%)	<0.001
CTDs (EDS/Marfan)	23 (1.7%)	8 (6.1%)	15 (1.2%)	<0.001
Extremity arterial dissection	28 (2.0%)	15 (11.5%)	13 (1.0%)	<0.001
Thoracic aortic dissection	19 (1.4%)	3 (2.3%)	16 (1.3%)	0.415
Dilated aorta *	191 (20.3%)	22 (23.4%)	169 (20.0%)	0.520
MVP	45 (3.3%)	7 (5.3%)	38 (3.0%)	0.249
Mitral regurgitation	143 (10.4%)	18 (13.7%)	125 (10.0%)	0.237
CTD Features				
Joint conditions	277 (20.1%)	32 (24.4%)	245 (19.6%)	0.233
Pectus deformities	14 (1.0%)	3 (2.3%)	11 (0.9%)	0.140
Valgus/varum deformities	6 (0.4%)	2 (1.5%)	4 (0.3%)	0.104
Scoliosis/kyphosis	15 (1.1%)	1 (0.8%)	14 (1.1%)	1.000
Varicose veins	24 (1.7%)	3 (2.3%)	21 (1.7%)	0.492
Organ perforation	73 (5.3%)	7 (5.3%)	66 (5.3%)	1.000
Outcomes				
Stroke	63 (4.6%)	16 (12.2%)	47 (3.8%)	<0.001
SCAD recurrence	615 (44.6%)	61 (46.6%)	554 (44.4%)	0.695
Death	15 (1.1%)	2 (1.5%)	13 (1.0%)	0.646

Continuous variables: mean ± SD, Welch’s *t*-test. Categorical variables: *n* (%), χ^2^ or Fisher’s exact test. * Dilated aorta data available in 939 patients (32% missing).

**Table 2:** Extracoronary Vascular Involvement: CACAD vs. SCAD-Only.

Variable	CACAD (*n* = 131)	SCAD-Only (*n* = 1249)	*p*-Value
FMD by Territory			
Any FMD	106 (80.9%)	458 (36.7%)	<0.001
Cervical FMD	88 (67.2%)	298 (23.9%)	<0.001
Carotid FMD	77 (58.8%)	268 (21.5%)	<0.001
Vertebral FMD	52 (39.7%)	165 (13.2%)	<0.001
Renal FMD	63 (48.1%)	290 (23.2%)	<0.001
Iliac/femoral FMD	41 (31.3%)	158 (12.7%)	<0.001
Non-Coronary Dissections			
Any non-coronary dissection	131 (100%)	35 (2.8%)	<0.001
Carotid dissection	80 (61.1%)	3 (0.2%)	<0.001
Vertebral dissection	51 (38.9%)	3 (0.2%)	<0.001
Extremity arterial dissection	15 (11.5%)	13 (1.0%)	<0.001
Aneurysms			
Any aneurysm	65 (49.6%)	163 (13.1%)	<0.001
Cerebral aneurysm	22 (16.8%)	44 (3.5%)	<0.001
Cervical aneurysm	33 (25.2%)	32 (2.6%)	<0.001
Renal aneurysm	9 (6.9%)	30 (2.4%)	0.008
Splenic aneurysm	10 (7.6%)	29 (2.3%)	0.001
Coronary aneurysm	4 (3.1%)	12 (1.0%)	0.057
Abdominal aortic aneurysm	2 (1.5%)	6 (0.5%)	0.172

FMD = fibromuscular dysplasia.

**Table 3:** Multivariable Logistic Regression—Predictors of CvAD.

Variable	OR (95% CI)	*p*-Value
Extremity arterial dissection	8.127 (3.412, 19.359)	<0.001
FMD	7.521 (4.674, 12.101)	<0.001
CTDs	6.848 (2.347, 19.982)	<0.001
Valgus/Varum	4.519 (0.655, 31.160)	0.126
Anxiety	1.687 (1.013, 2.808)	0.044
Migraine	1.498 (0.932, 2.410)	0.095
Pectus Deformities	1.494 (0.307, 7.258)	0.619
MVP	1.335 (0.497, 3.585)	0.566
Gender (Female)	1.070 (0.458, 2.501)	0.875
Joint Conditions	0.945 (0.560, 1.596)	0.833
Mitral Regurgitation	0.943 (0.488, 1.822)	0.861

Model *n* = 1380; 131 events; pseudo-R^2^ = 0.170; model *p* < 0.001. Variables have *p* < 0.20 in univariate analysis plus gender (clinically mandated). OR = odds ratio; CI = confidence interval.

**Table 4:** Multivariable Logistic Regression—Predictors of Stroke.

Variable	OR (95% CI)	*p*-Value
CvAD	6.373 (2.928, 13.873)	<0.001
TAD	4.258 (0.909, 19.947)	0.066
Atrial Fibrillation	2.668 (1.202, 5.921)	0.016
Obesity	2.684 (1.306, 5.517)	0.007
Depression	1.999 (0.979, 4.081)	0.057
Hypertension	1.778 (0.933, 3.391)	0.080
Hyperlipidemia	1.765 (0.883, 3.526)	0.108
MR	1.451 (0.664, 3.171)	0.350
Migraine	1.361 (0.716, 2.590)	0.347
CTDs	1.355 (0.278, 6.602)	0.707
Organ Perforation	1.271 (0.526, 3.070)	0.595
Heart Failure	1.187 (0.612, 2.305)	0.612
Age	1.000 (0.974, 1.028)	0.972
Joint Conditions	0.799 (0.400, 1.594)	0.523
Diabetes	0.767 (0.301, 1.959)	0.580
Anxiety	0.732 (0.361, 1.484)	0.387
SCAD Recurrence	0.628 (0.345, 1.141)	0.126
FMD	0.375 (0.184, 0.766)	0.007

Model *n* = 1380; 63 events; pseudo-R^2^ = 0.183; model *p* < 0.001. CvAD = cervical arterial dissection; TAD = thoracic aortic dissection; FMD = fibromuscular dysplasia; MR = mitral regurgitation.

**Table 5:** CvAD as a Predictor of Clinical Outcomes.

Outcome	CACAD	SCAD-Only	Adjusted OR (95% CI)	*p*-Value
Stroke	16 (12.2%)	47 (3.8%)	6.373 (2.928, 13.873)	<0.001
All-cause mortality	2 (1.5%)	13 (1.0%)	2.987 (0.472, 18.920)	0.245
SCAD recurrence	61 (46.6%)	554 (44.4%)	0.869 (0.587, 1.287)	0.483

Adjusted ORs derived from separate multivariable logistic regression models for each outcome. Full models available in Supplemental Tables S2 and S3.

## Data Availability

The data presented in this study are available on request from the corresponding author. The data are not publicly available due to patient privacy restrictions.

## Supplementary Materials

The following supporting information can be downloaded at: https://www.mdpi.com/article/10.3390/jcm15135302/s1. Table S1: Univariate Logistic Regression Screening for CvAD; Table S2: Full Multivariable Model—Predictors of All-Cause Mortality; Table S3: Full Multivariable Model—Predictors of SCAD Recurrence.

## Abbreviations

The following abbreviations are used in this manuscript:

SCAD	Spontaneous coronary artery dissection
CvAD	Cervical artery dissection
CACAD	Combined coronary and cervical arterial dissection
FMD	Fibromuscular dysplasia
CTD	Connective tissue disorder
ACS	Acute coronary syndrome
MVP	Mitral valve prolapse
TAD	Thoracic aortic dissection
MR	Mitral regurgitation
POTS	Postural orthostatic tachycardia syndrome
OR	Odds ratio
CI	Confidence interval
