# Supplementary material for: Prevalence, Predictors, and Clinical Outcomes of Cervical Arterial Dissection in Patients with Spontaneous Coronary Artery Dissection: A Multicenter Retrospective Cohort Study
Source: J Clin Med. 2026 Jul 7;15(13):5302. doi: 10.3390/jcm15135302 (PMC13362897; doi:10.3390/jcm15135302)
Supplement: Supplementary file 1 [file jcm-15-05302-s001.zip › jcm-4383923-supplementary.pdf]

SUPPLEMENTAL TABLES

Supplemental Table S1. Univariate Logistic Regression Screening for CvAD.

Variable	OR	95% CI	p-value	N
Extremity Dissection	12.294	5.711–26.466	<0.001	1380
FMD	7.323	4.666–11.493	<0.001	1380
CTDs (EDS/Marfan)	5.351	2.224–12.872	<0.001	1380
Pectus Deformities	2.638	0.726–9.578	0.140	1380
MVP	1.799	0.787–4.114	0.164	1380
Migraine	1.695	1.112–2.584	0.014	1380
Valgus/Varum	4.826	0.875–26.602	0.071	1380
Anxiety	1.434	0.955–2.155	0.082	1380
Mitral Regurgitation	1.432	0.843–2.435	0.184	1380
Female Sex	1.353	0.643–2.845	0.426	1380
Joint Conditions	1.325	0.868–2.021	0.192	1380
Dilated Aorta	1.222	0.737–2.028	0.437	939
Depression	1.231	0.758–1.999	0.402	1380
Atrial Fibrillation	1.130	0.531–2.405	0.752	1380
Hyperlipidemia	1.114	0.770–1.610	0.567	1380
Age (at Diagnosis)	1.005	0.989–1.022	0.523	1380
Hypertension	0.941	0.641–1.380	0.754	1380
Heart Failure	0.928	0.564–1.530	0.771	1380
TAD	1.806	0.519–6.282	0.353	1380
Flat Feet	1.921	0.416–8.862	0.403	1380
Thyroid Disorders	0.719	0.396–1.304	0.277	1380
Obesity	0.726	0.382–1.380	0.329	1380
Scoliosis/Kyphosis	0.679	0.089–5.202	0.709	1380
Diabetes Mellitus	0.630	0.250–1.588	0.327	1380
Dermatological Conditions	0.526	0.070–3.973	0.533	1380

Variables with $p < 0.20$ were included in the multivariable model (Table 3).

Supplemental Table S2. Full Multivariable Model — Predictors of All-Cause Mortality.

Variable	OR (95% CI)	p-value
CTDs	16.038 (1.370, 187.815)	0.027
Diabetes Mellitus	4.357 (1.207, 15.734)	0.025
Atrial Fibrillation	4.006 (1.062, 15.106)	0.040
Anxiety	3.080 (0.761, 12.463)	0.115
CvAD	2.987 (0.472, 18.920)	0.245
Heart Failure	2.914 (0.801, 10.608)	0.105
Thyroid	1.376 (0.389, 4.862)	0.620
Age	1.073 (1.016, 1.134)	0.012
MR	1.019 (0.257, 4.039)	0.979
Hypertension	0.994 (0.253, 3.906)	0.994
Depression	0.968 (0.239, 3.919)	0.964
Hyperlipidemia	0.932 (0.179, 4.865)	0.933
Obesity	0.916 (0.220, 3.814)	0.904
Joint Conditions	0.748 (0.188, 2.980)	0.681
Scoliosis	0.622 (0.034, 11.242)	0.748
FMD	0.402 (0.086, 1.881)	0.247
SCAD Recurrence	0.134 (0.016, 1.118)	0.063

Model n = 1380; 15 events; pseudo-R^2 = 0.319; model $p < 0.001$. Note: With only 15 events and 17 predictors, this model exceeds the 10-events-per-variable guideline and should be interpreted cautiously.

Supplemental Table S3. Full Multivariable Model — Predictors of SCAD Recurrence.

Variable	OR (95% CI)	p-value
Migraine	2.255 (1.660, 3.065)	<0.001
FMD	1.595 (1.259, 2.019)	<0.001
Gender	1.350 (0.877, 2.078)	0.172
Anxiety	0.728 (0.520, 1.018)	0.064
CvAD	0.869 (0.587, 1.287)	0.483
Age	0.998 (0.987, 1.010)	0.783
Hyperlipidemia	0.957 (0.733, 1.250)	0.749
Joints	0.899 (0.636, 1.271)	0.547
Hypertension	0.864 (0.661, 1.129)	0.285
Obesity	0.777 (0.497, 1.213)	0.266
Heart Failure	0.744 (0.532, 1.040)	0.083
Organ Perforation	0.961 (0.556, 1.662)	0.887
MR	0.676 (0.461, 0.993)	0.046
Stroke	0.666 (0.370, 1.200)	0.176
Dermatological Conditions	0.657 (0.217, 1.985)	0.456
Scoliosis	0.550 (0.160, 1.887)	0.342
Diabetes Mellitus	0.450 (0.250, 0.813)	0.008
Depression	1.088 (0.744, 1.591)	0.664

Model n = 1380; 615 events; pseudo-R^2 = 0.051; model $p < 0.001$.
